# The MAGENTA model for individual prediction of in-hospital mortality in chronic obstructive pulmonary disease with acute exacerbation in resource-limited countries: A development study

**DOI:** 10.1371/journal.pone.0256866

**Published:** 2021-08-27

**Authors:** Prachya Mekanimitdee, Thotsaporn Morasert, Jayanton Patumanond, Phichayut Phinyo

**Affiliations:** 1 Department of Internal Medicine, Surat Thani Hospital, Surat Thani, Thailand; 2 Pulmonary and Critical Care Medicine, Department of Internal Medicine, Surat Thani Hospital, Surat Thani, Thailand; 3 Center for Clinical Epidemiology and Clinical Statistics, Faculty of Medicine, Chiang Mai University, Chiang Mai, Thailand; 4 Department of Family Medicine, Faculty of Medicine, Chiang Mai University, Chiang Mai, Thailand; 5 Musculoskeletal Science and Translation Research (MSTR) Cluster, Chiang Mai University, Chiang Mai, Thailand; University of California, Davis, UNITED STATES

## Abstract

**Background:**

Acute exacerbation of chronic obstructive pulmonary disease (AECOPD) is a common undesirable event associated with significant morbidity and mortality. Several clinical prediction tools for predicting in-hospital mortality in patients with AECOPD have been developed in the past decades. However, some issues concerning the validity and availability of some predictors in the existing models may undermine their clinical applicability in resource-limited clinical settings.

**Methods:**

We developed a multivariable model for predicting in-hospitality from a retrospective cohort of patients admitted with AECOPD to one tertiary care center in Thailand from October 2015 to September 2017. Multivariable logistic regression with fractional polynomial algorithms and cluster variance correction was used for model derivation.

**Results:**

During the study period, 923 admissions from 600 patients with AECOPD were included. The in-hospital mortality rate was 1.68 per 100 admission-day. Eleven potential predictors from the univariable analysis were included in the multivariable logistic regression. The reduced model, named MAGENTA, incorporated seven final predictors: age, body temperature, mean arterial pressure, the requirement of endotracheal intubation, serum sodium, blood urea nitrogen, and serum albumin. The model discriminative ability based on the area under the receiver operating characteristic curve (AuROC) was excellent at 0.82 (95% confidence interval 0.77, 0.86), and the calibration was good.

**Conclusion:**

The MAGENTA model consists of seven routinely available clinical predictors upon patient admissions. The model can be used as an assisting tool to aid clinicians in accurate risk stratification and making appropriate decisions to admit patients for intensive care.

## Introduction

Chronic obstructive pulmonary disease (COPD) has progressively become a global public health concern [1]. The disease is commonly defined as a progressive and persistent airflow limitation, which is associated with a chronic inflammatory response of the respiratory tract and the lung parenchyma to noxious particles or gases [2]. Despite being claimed as a preventable and controllable disease, COPD was consistently reported as the leading cause of death among other chronic diseases [3]. Patients with COPD often experience an acute, undesirable worsening of clinical condition, generally described as an acute exacerbation of COPD (AECOPD) [4], which usually requires subsequent therapy. The occurrence of AECOPD substantially affects the patient’s quality of life, the decline in health status and pulmonary function, the COPD progression, the risk of further exacerbation episodes, the number of hospitalizations and readmissions, and, most importantly, the patient mortality [4–6]. Previously, the estimated incidences and in-hospital mortality of AECOPD varied across studies from 0.65 to 1.40 person-years [7–9] and from 2.5% to 25% [10–12], respectively.

Continuous efforts have been made to reduce the in-hospital morbidity and mortality for AECOPD patients. One approach is to apply clinical prediction tools for making sound clinical decisions based on predicted risk of in-hospital death towards appropriate patient disposition (early supported hospital discharge for low-risk patients, general ward admission or intensive care unit admission for medium to high-risk patients), early aggressive treatment, and initiation of end-of-life care for high-risk patients [13]. Several clinical tools for the prediction of in-hospital mortality, either COPD-specific or non-specific, have been developed and validated in patients with AECOPD [14], such as the Acute Physiology and Chronic Health Evaluation (APACHE) II [15], Simplified Acute Physiology Score (SAP) II [16], BAP-65 (Blood urea nitrogen, Altered mental status, Pulse, Age ≥65) [17], CURB-65 (Confusion, blood Urea nitrogen, Respiratory rate, Blood pressure, Age ≥65) [18], and DECAF (Dyspnea, Eosinopenia, Consolidation, Acidemia, Atrial Fibrillation) score [13]. Among all available decision tools, the DECAF score was superior to other scores in predicting short-term mortality in patients with AECOPD [19].

Even though the DECAF score carries excellent discriminative performance, both in the derivation (area under the receiver operating characteristic curve (AuROC) 0.86) and the validation datasets (AuROC 0.83) [13, 19], some specific issues regarding its predictors may undermine the clinical applicability of the score in some clinical setting. First, the DECAF score calculation essentially requires measurements of extended Medical Research Council Dyspnea score or eMRCD, which was rarely measured in routine practice in Thailand and presumably other developing countries with high clinical workloads. Second, an arterial puncture was also required for the evaluation of acidemia. In Thailand, blood gas analysis was only performed in patients with AECOPD who were intubated and needed regular ventilation adjustment [20]. Thus, for patients with AECOPD without acute respiratory failure, blood gas analysis was not generally available for the calculation of the DECAF score. Third, using blood eosinophil count as a predictor raised concerns when applied in countries with a high prevalence of parasitic infestation, such as in Thailand [21] and Asian countries [22].

In this study, we aimed to develop a novel clinical prediction tool that incorporates routinely available clinical parameters for predicting in-hospital mortality for patients with AECOPD, which would be more practical and generalizable to most health care settings than the previously developed scoring system, especially in the developing countries.

## Materials and methods

### Study design

Prognostic model research was conducted with a retrospective cohort design. All data were obtained from electronic medical records of AECOPD patients admitted to Surat Thani Hospital between October 2015 and September 2017. The Institutional Review Board and the Ethics Committee of Surat Thani Hospital approved the study protocol (Approval ID. 25/2563). Due to the retrospective nature of data collection, informed consent is waived by the ethics committee. All the data used in this study were fully anonymized before the statistical analysis was conducted. The patients’ medical records were accessed between January 2020 and April 2020. We followed the Transparent Reporting of a multivariable prediction model for Individual Prognosis Or Diagnosis (TRIPOD) statements for study conduct and reporting [23].

### Setting

Surat Thani Hospital is a university-affiliated tertiary care and referral center located in the southern part of Thailand. Although the hospital carries a total capacity of 1,000 beds, there are only 50 beds for medical intensive care. Regarding the chain of care for AECOPD patients, emergency department physicians are the first to encounter the patients. After an initial evaluation, emergency physicians must decide whether to discharge or admit the patient for the continuation of care. It is undeniable that severe AECOPD patients with acute respiratory failure require admission to the medical intensive care unit (MICU) for close monitoring and early aggressive treatment. However, as the number of available beds in the MICU is usually limited, several intubated AECOPD patients must be admitted to general wards. Patient risk stratification is used to prioritize the patient for admission to the MICU by an attending physician upon admission to the wards.

Although the recent guideline recommends bilevel non-invasive ventilation (NIV) as the initial ventilatory support for AECOPD patients with acute respiratory failure [24], NIV was not widely available and routinely performed in our center during the study period. Besides, many AECOPD patients with acute respiratory failure were intubated and referred from community hospitals within the catchment area, where NIV was not available.

### Study domain

We defined the study domain as patients intended to be prognosticated with the prediction model, which were AECOPD patients who required hospitalization in Surat Thani Hospital during the study period. AECOPD diagnosis was based on the International Statistical Classification of Disease and Related Health Problems 10 (ICD-10) codes J44.0, J44.1, and J44.9. These ICD-10 codes must be included as the principal diagnosis for that admission record to be eligible for inclusion. All eligible records underwent a thorough review by certified pulmonologists. We excluded patients with spirometry results that were not consistent with COPD diagnosis or patients with other active pulmonary diseases, such as lung cancer, acute pulmonary embolism, and acute respiratory distress syndrome). As we intended to compare the performance of the newly derived model to the conventional scoring system, admission records without the data on CURB-65 evaluation were excluded. Recurrent exacerbation episodes requiring hospitalization of the same patient were not excluded, as the observation unit was each admission record, not an individual patient.

### Study endpoint

The clinical endpoint to be predicted was in-hospital mortality, which was based on the documented survival status of each patient admission in the hospital discharge summary. All included admission records were categorized into non-survived and survived admissions.

### Data collection

Patient clinical profiles upon their admissions to the hospital, the point of prediction, were extracted. Data on demographic characteristics (age and gender), anthropometric characters (weight, height, and body mass index (BMI)), smoking status, presence of comorbidity, COPD status (spirometry results, use of long-term oxygen therapy, Cor pulmonale, and history of emergency department visit or hospitalization within the past year), medications used, and influenza vaccination were collected. We also collected the data on clinical parameters, including initial vital signs (e.g., body temperature (BT), heart rate (HR), respiratory rate (RR), systolic blood pressure (SBP), diastolic blood pressure (DBP), and mean arterial pressure (MAP), initial status (e.g., respiratory failure), initial investigations (e.g., complete blood count, blood chemistry, chest radiography), and point of care glucose testing. Any variable with more than 50% missing data was not selected as a candidate predictor.

### Candidate predictors

Among all collected clinical parameters at baseline, we preselected twelve candidate predictors based on the availability of data, clinical knowledge, and extensive review of clinical evidence as follow: age [25], BT [20], MAP [26], requiring endotracheal intubation due to respiratory failure [27], the presence of radiographic consolidation [28], white blood cell (WBC) count [29], eosinophil count [13], serum sodium (Na) [30], blood urea nitrogen (BUN) [31], serum creatinine (SCr) [32], serum albumin [30], and point of care glucose level [33]. Although baseline dyspnea score level, eMRCD, was previously identified as a significant predictor of AECOPD mortality [34], it was not included in statistical modeling as the parameter was not routinely evaluated and recorded in our practice.

### Statistical analysis

For the description of categorical data, frequency and percentage were used. Mean and standard deviation (SD) was used for normally distributed continuous data, whereas median (50^th^ percentile or P_50_) and interquartile range (IQR) was used for skewed continuous data. The data on preselected clinical predictors from non-survived and survived admissions were compared using an independent t-test for normally distributed continuous data, Mann-Whitney U-test for skewed continuous data. Fisher’s exact probability test was used to compare the difference of categorical data between the two groups. A two-sided p-value <0.05 was considered statistically significant. Statistical analyses were performed using Stata 16.

#### Prognostic model development

For continuous predictors, multivariable fractional polynomials or MFP algorithms were used to identify the optimal fractional polynomial transformation of each predictor to be fitted in a binary logistic model [35]. For missing data, multiple imputation with chained equation (MICE) was used. Age, gender, MAP, radiographic consolidation, requirement of endotracheal intubation, BUN, SCr, and survival status (the study endpoint) were used as independent variables in predictive mean matching (PMM) methods with K-nearest neighbor where *k* = 10. A total of 35 imputed datasets was derived during the imputation procedures. The number of imputed datasets was based on a variable with the highest percentage of missing values.

Significant candidate clinical predictors from the univariable analysis were included in a multivariable logistic regression model with cluster variance correction to account for correlated admission records within the same patient [36]. We first employed the full model approach by simultaneously including all candidate predictors within the model. Then, backward elimination was done to remove non-significant (p-value>0.05) and non-contributing factors from the model, yielding the reduced model. A sensitivity analysis was performed by including only one admission visit per patient (excluding readmission records) to eliminate the potential correlation issue.

#### Evaluation of model performance and internal validation

The model performance was measured in terms of discrimination and calibration. The model discriminative performance was quantified using the AuROC. As we did not have the essential data on the predictors of the DECAF score, the discriminative ability of the newly derived model in predicting mortality was compared to the CURB-65 score, which was previously reported to have prognostic utility in predicting mortality in AECOPD [37]. The model calibration was visualized with a calibration plot to examine the agreement of the model predicted probabilities and the observed proportions of event occurrence. We also performed Hosmer-Lemeshow goodness-of-fit statistical testing for the model calibration.

According to the TRIPOD statement [23], resampling techniques (e.g., cross-validation and bootstrapping) are recommended over the widely-used random split-sampling techniques. In this study, internal validation of the derived prognostic model was performed with a bootstrapping procedure with 100 replicates. Bootstrap resampling allowed us to use all available data during the model derivation and enabled the estimation of shrinkage factor to be used during external validation. Apparent AuROC, test AuROC, mode optimism (the difference between apparent and test AuROC), and the calibration slope (shrinkage factor) were estimated and reported.

#### Model presentation and evaluation of prognostic accuracy

The derived prognostic model was presented as a logistic regression equation. The predicted probability of in-hospital mortality can be calculated by the inverse logit transformation of the estimated linear predictor (z) as follows: probability = e^z^/(1+e^z^), where e is the base value of natural logarithms. The predicted probabilities were then categorized into three risk groups for clinical applicability, which were low risk, intermediate risk, and high risk. We evaluated the prognostic accuracy of each categorized risk group by estimating group specific-sensitivity, -specificity, and -positive likelihood ratio. The appropriate cutoff points for each risk group were identified during statistical analysis based on data distribution, positive likelihood ratios, and the consensus of the investigators.

An online web application of the model is available at: https://www.calconic.com/calculator-widgets/copd-magenta/5f34d4d3a2d88c002959d229.

## Results

**Fig 1:**
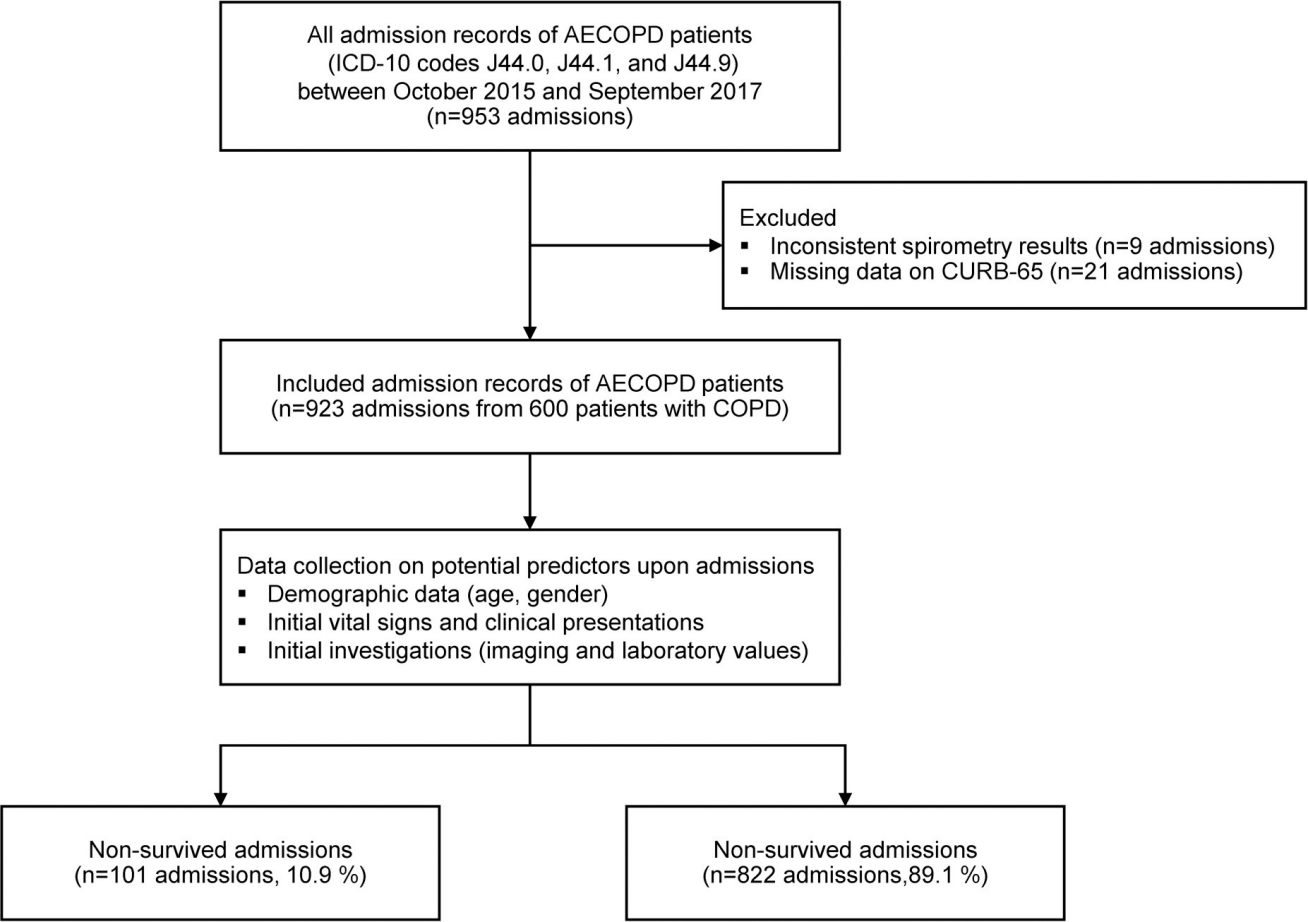
Study flow diagram of patient cohort.

From October 2015 to September 2017, 953 admission records of AECOPD patients were retrieved and reviewed for eligibility. Thirty were excluded (9 admissions with spirometry results not consistent with COPD diagnosis and 21 admissions with missing data on CURB-65 evaluation) (Fig 1).

### Characteristics of AECOPD admissions

Nine-hundred and twenty-three admissions due to AECOPD of 600 patients were included for statistical analysis. Of these numbers, there were 101 (10.9%) non-survived admissions and 822 (89.1%) survived admissions. The in-hospital mortality rate was 1.68 per 100 admission-day (101/5996 admission-day). Only 95 (10.3%) admission records were directed to the MICUว the rest were admitted to general medical wards (828, 89.7%). The median length of hospital stay was 4 days (IQR 2, 7 days) for general medical wards and 5 days (IQR 3, 9 days) for ICU admissions.

### Characteristics of patients with COPD

Most patients with COPD included in this study were male (515, 85.8%) with a mean age of 74 years (SD ±11, range 42–99). Only 14.3% were active smokers with a median smoking pack-year of 20 (IQR 11, 30). Almost 80% of the patients had at least one medical comorbidity. The most common was hypertension (78.0%), diabetes mellitus (11.3%), and ischemic heart disease (8.8%). Spirometry results were available for assessing disease severity in only 127 (21.2%) patients. Detailed clinical characteristics, including COPD severity of included patients, are shown in Table 1. S1 and S2 Tables show the comparison of clinical characteristics between patients admitted to the MICU and patients admitted to general medical wards, overall patients, and intubated patients, respectively.

**Table 1:** Characteristics of COPD patients with acute exacerbation requiring hospitalization (n = 600 patients).

Characteristics	Missing values, n(%)	Patients (n = 600)
**Male**, (n, %)	0 (0)	515 (85.8)
**Age**, years, mean (±SD)	0 (0)	74.1 (±11.1)
**Body mass index**, kg/m^2^, mean (±SD)	281 (46.8)	20.3 (±4.3)
**Smoking status**		
Never smoker, (n, %)	27 (4.5)	44 (7.3)
Former smoker, (n, %)		443 (73.8)
Active smoker, (n, %)		86 (14.3)
Pack-year, median (IQR)	231 (38.5)	20 (11, 30)
**Underlying diseases**, (n, %)		
Present (any)	0 (0)	468 (78.0)
Hypertension	0 (0)	253 (42.2)
Diabetes mellitus	0 (0)	68 (11.3)
Ischemic heart disease	0 (0)	53 (8.8)
Atrial fibrillation	0 (0)	34 (5.7)
Left ventricular dysfunction	0 (0)	14 (2.3)
Chronic kidney disease	0 (0)	51 (8.5)
Cerebrovascular disease	0 (0)	48 (8.0)
Cognitive impairment	0 (0)	5 (0.8)
**COPD status**		
FEV1/FVC ratio, mean (±SD)	473 (78.8)	0.49 (±0.11)
FEV1, % predicted, median (IQR)	473 (78.8)	37 (29, 53)
FVC, % predicted, median (IQR)	473 (78.8)	64 (51, 83)
GOLD severity of airflow limitation, (n, %)		
FEV1 ≥80% predicted (stage I)	473 (78.8)	6 (1.0)
FEV1 50–79% predicted (stage II)		29 (4.8)
FEV1 30–49% predicted (stage III)		59 (9.8)
FEV1 <30% predicted (stage IV)		33 (5.5)
Long-term oxygen therapy, (n, %)	0 (0)	29 (4.8)
Cor pulmonale, (n, %)	0 (0)	25 (4.2)
ED visit due to AECOPD within the past year, (n, %)		
0	0 (0)	404 (67.3)
1		95 (15.8)
≥2		101 (16.8)
Hospitalization due to AECOPD within the past year, (n, %)		
0	0 (0)	485 (80.8)
1		81 (13.5)
≥2		34 (5.7)
**Inhaled controller medications**, (n, %)		
Present (any)	0 (0)	344 (57.3)
Salmeterol/fluticasone	3 (0.5)	294 (49.0)
Formoterol/budesonide	3 (0.5)	8 (1.3)
Tiotropium	3 (0.5)	65 (10.8)
Budesonide	3 (0.5)	31 (5.2)
**Influenza Vaccination**	0 (0)	37 (6.2)

**Abbreviations:** AECOPD, acute exacerbation of chronic obstructive pulmonary disease; COPD, chronic obstructive pulmonary disease; ED, emergency department; FEV1, forced expiratory volume-one second; FVC, forced vital capacity; GOLD, global initiative for chronic obstructive pulmonary disease; IQR, interquartile range; SD, standard deviation.

### Candidate clinical predictors

The comparisons of 12 pre-selected candidate clinical predictors between non-survived and survived admissions of AECOPD patients are shown in Table 2. According to the univariable analysis, it was evident that patients who did not survive the admission had significantly higher age, higher BT, lower MAP, higher WBC counts, lower eosinophil counts, lower serum Na level, higher BUN level, higher SCr, and lower serum albumin than those of patients who did survive the admission (Table 2). Non-survived admissions also had significantly higher proportion of intubation (95.1% vs. 68.3%, p<0.001) and radiographic consolidation (64.4% vs. 37.0%, p<0.001). However, the initial point-of-care glucose levels were not significantly differed between groups (P_50_ 143 (IQR 108, 193) vs. P_50_ 142 (IQR 118, 169), p = 0.528) and were omitted from subsequent multivariable modeling.

**Table 2:** Pre-specified candidate clinical predictors of non-survived and survived admissions of AECOPD patients (n = 923 admissions).

Characteristics	Missing values, n(%)	Non-survived admissions (n = 101)		Survived admissions (n = 822)		p-value
		mean	±SD	mean	±SD	
**Demographic data**						
Age, years	0 (0)	76.8	±11.0	74.1	±11.1	0.020
**Initial assessments**						
BT,°C	1 (0.1)	37.4	±0.9	37.1	±0.6	<0.001
MAP, mmHg	0 (0)	90.3	±20.3	98.3	±15.4	<0.001
Require intubation, (n, %)	0 (0)	96	(95.1)	561	(68.3)	<0.001
**Initial investigations**						
Radiographic consolidation, (n, %)	0 (0)	65	(64.4)	304	(37.0)	<0.001
**Complete blood count**						
WBC count, /mm^3^	1 (0.1)	15296	±6978	13738	±6083	0.017
Eosinophil count, /mm^3^, median (IQR)	1 (0.1)	9.5	(0, 172)	40.8	(0, 228)	0.010
**Blood chemistry**						
Sodium, mmol/l	4 (0.4)	137.5	±7.6	138.8	±4.6	0.013
BUN, mg/dl, median (IQR)	0 (0)	21	(14, 32)	15	(11, 21)	<0.001
SCr, mg/dl, median (IQR)	0 (0)	1.1	(0.8, 1.5)	0.9	(0.8, 1.2)	0.002
Serum albumin, g/dl	323 (35.0)	3.4	±0.6	3.9	±0.5	<0.001
**Point of care testing**						
Initial glucose, mg/dl	218 (23.6)	143	(108, 193)	142	(118, 169)	0.528

**Abbreviations:** BT, body temperature; BUN, blood urea nitrogen; IQR, interquartile range; MAP, mean arterial pressure; SCr, serum creatinine; SD, standard deviation; WBC, white blood cell.

### Derivation of prognostic model

Eleven significant clinical predictors from the univariable analysis were included in the multivariable logistic regression to derive the prognostic model: age, BT, MAP, endotracheal intubation, radiographic consolidation, WBC count, eosinophil count, serum Na, BUN, SCr, and serum albumin. The MFP procedure was performed to identify the optimal transformation of non-linear predictor-outcome associations for the modeling of continuous predictors. With the full model approach, five out of eleven predictors were found to be independent predictors of in-hospital mortality: BT, MAP, endotracheal intubation, serum Na, and serum albumin. After the backward elimination of non-significance predictors, BUN was shown as another significant predictor of mortality. Age was included in the reduced model regardless of statistical significance due to its clinical importance. The optimal fractional polynomials transformation of continuous predictors, logit regression coefficients with 95% confidence interval, and p-values are presented in Table 3. The sensitivity analysis results are presented online in S3 Table.

**Table 3:** Fractional polynomial transformation (of continuous parameters). Odds ratios from full and reduced multivariable logistic model (with cluster variance correction) for predictors of in-hospital mortality of AECOPD patients (n = 923 admission visits). Missing data on predictors were imputed with multiple imputation with chained equation (partial mean matching).

Parameters		Full model				Reduced model			
	Optimal FP transformations	OR	95% CI	P-value	ß	OR	95% CI	P-value	ß
**Demographic data**									
Age, years	Age-74.3651	1.01	0.99, 1.04	0.375	0.0116	1.01	0.98, 1.03	0.490	0.0084
**Initial assessments**									
BT,°C	BT-37.1226	1.83	1.33, 2.51	<0.001	0.6032	1.91	1.38, 2.63	<0.001	0.6452
MAP, mmHg	(MAP/100)^-2^–1.0545	2.39	1.53, 3.72	<0.001	0.8716	2.36	1.51, 3.70	<0.001	0.8591
Require intubation	Original binary form	8.70	3.04, 24.87	<0.001	2.1631	8.59	3.18, 23.15	<0.001	2.1500
**Initial investigations**									
Radiographic consolidation	Original binary form	1.51	0.91, 2.50	0.113	0.4098	Not included			
**Complete blood count**									
WBC count, /mm^3^	WBC-13910.8931	1.00	0.99, 1.00	0.242	0.0001	Not included			
Eosinophil count, /mm^3^	Eosinophil-223.0664	1.00	0.99, 1.00	0.365	0.0002	Not included			
**Blood chemistry**									
Na, mmol/l	Na-138.6417	0.95	0.91, 0.99	0.035	-0.0464	0.95	0.91, 0.99	0.031	-0.0473
BUN, mg/dl	BUN-18.7486	1.01	0.99, 1.03	0.200	0.0130	1.02	1.01, 1.03	0.007	0.0199
SCr, mg/dl	SCr-1.077	1.26	0.79, 1.99	0.331	0.2279		Not included		
Serum albumin, g/dl	Alb-3.8174	0.37	0.21, 0.64	<0.001	-1.0022	0.35	0.20, 0.59	<0.001	-1.0573
Constant					-4.6757				-4.4415
AuROC		0.82	0.78, 0.87			0.82	0.77, 0.86		

**Abbreviations:** AuROC, area under the receiver operating characteristic curve; ß, beta-coefficient (log odds ratio); BT, body temperature; BUN, blood urea nitrogen; CI, confidence interval; FP, fractional polynomial; MAP, mean arterial pressure; NA, not applicable; Na, serum sodium; OR, odds ratio; SCr, serum creatinine; WBC, white blood cell.

### Test of model performance

**Fig 2:**
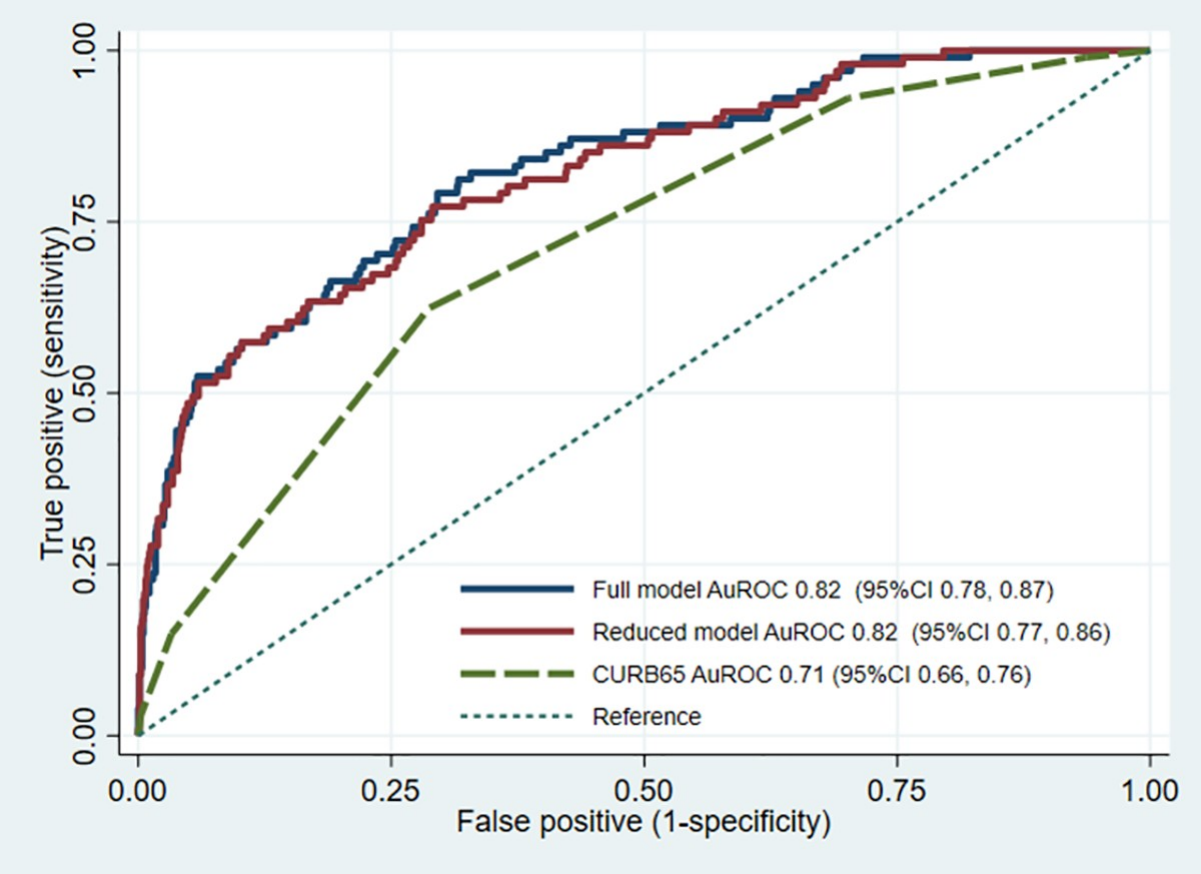
Receiver operating characteristic curve. Discriminative ability (area under the receiver operating characteristic curves) of the derived prediction models (full and reduced models) and the conventional CURB-65 score.

Both the full model (11 predictors) and the reduced (7 predictors) prognostic model showed excellent discriminative ability with an AuROC of 0.82 (95% confidence interval (CI) 0.78, 0.87) and 0.82 (95%CI 0.77, 0.86), respectively. Compared to the conventional CURB-65 scoring, our newly derived prognostic models, both the full and the reduced model, showed a significantly higher discriminative performance for prediction of in-hospital mortality in AECOPD patients (0.82 vs. 0.72, p<0.001 and 0.82 vs. 0.72, p<0.001, respectively) (Fig 2).

**Fig 3:**
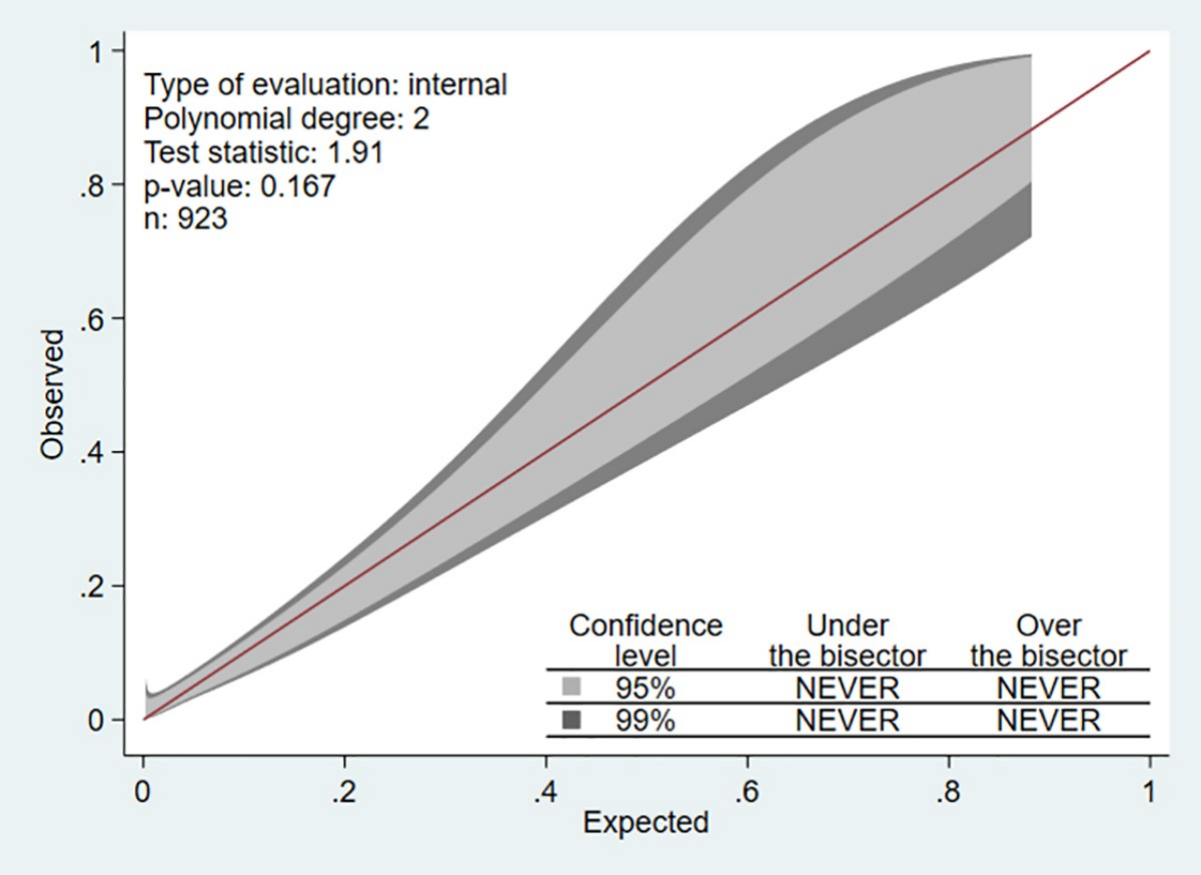
Calibration plot. Visualizing agreement between model predicted and observed proportion of in-hospital mortality in AECOPD patients.

We focused mainly on the reduced model, as the model carried fewer predictors with comparable performance to the full model. The discriminative ability was preserved in the subgroup that was admitted to general medical wards (AuROC 0.84, 95%CI 0.79, 0.89), whereas the ability dropped in the subgroup that was admitted to the ICUs (AuROC 0.68, 95%CI 0.54, 0.83). The reduced model showed good internal calibration according to the calibration plot (Fig 3). The Hosmer-Lemeshow goodness-of-fit statistics was insignificant (p = 0.062). Internal validation with bootstrapping procedure with 200 replicants showed an apparent AuROC of 0.82 (range 0.75 to 0.88) and a test AuROC of 0.81 (range 0.80 to 0.82). The optimism of AuROC was 0.010 (range -0.054 to 0.078). The estimated average calibration slope, or the shrinkage factor, was 0.95 (range 0.67 to 1.25).

### Model prognostic accuracy

The predicted probabilities of in-hospital mortality can be calculated with the following equation: probability = e^z^/(1+e^z^), where z = -4.4415+ 0.0084(age-74.3651) + 0.6452(BT-37.1226 + 0.8591((MAP/100)^-2^–1.0545) + 2.15(requiring intubation) + -0.0473(serum Na-138.6417) + 0.0199(BUN-18.7486) + -1.0573(serum albumin-3.8174).

For the clinical applicability of the model, the predicted probabilities were categorized into three clinical risk groups as follows: low-risk (<5.0%), intermediate-risk (5.0–14.9%), and high-risk (≥15%). The sensitivity, specificity, and positive likelihood ratios of each categorized risk group are presented in Table 4.

**Table 4:** Prognostic accuracy of the model for prediction of in-hospital mortality of AECOPD patients.

Predicted probability	Not survived	Survived	PPV% (95%CI)	LR+ (95%CI)	Interpretation
<5.0%	11	364	2.93 (1.47, 5.19)	0.25 (0.12, 0.47)	Low risk
5.0–14.9%	28	325	7.93 (5.34, 11.26)	0.70 (0.44, 1.10)	Intermediate risk
≥15.0%	62	133	31.79 (25.32, 38.83)	3.79 (2.58, 5.54)	High risk
Total	101	822			

**Abbreviations:** CI, confidence interval; PPV, positive predictive value; LR+, positive likelihood ratio.

## Discussion

This study developed the novel prediction model as a bedside, web-based application for predicting in-hospital mortality among AECOPD patients to aid clinicians in accurate risk stratification and making appropriate decisions to admit patients for intensive care. Our final model comprised seven routinely available predictors upon patient admissions: **M**ean arterial pressure, **A**ge, blood urea nitro**G**en, **E**ndotracheal intubation, serum sodium (**N**a), body **T**emperature, and serum **A**lbumin, hence the name “MAGENTA.” The model showed good to excellent discriminative performance and was well calibrated during internal validation.

Our cohort of AECOPD patients was derived from a single tertiary care setting in the southern part of Thailand. The in-hospital mortality rate of the cohort was approximately 11%, which was similar to other studies [13, 25, 38], including the cohort from which the DECAF score was developed. As our cohort included both AECOPD patients from ICU and non-ICU settings, the mortality rate was much lower than that of one study (38%), including only patients in the ICU [39]. Due to disproportional numbers of ICU beds to the number of patients, some mechanically ventilated AECOPD patients had to be operated in general medical wards rather than ICU. Unfortunately, this unsatisfactory situation was a common occurrence in most tertiary care centers in Thailand and many low to middle-income countries [40, 41]. Therefore, in these resource-limited settings, a clinical tool that properly predicts in-hospital mortality would help clinicians triage AECOPD patients for ICU admissions or guide early invasive treatment for high-risk patients.

The MAGENTA score consists of seven independent factors for the prediction of in-hospital mortality in AECOPD patients. The ability of each clinical predictor, including intubation due to respiratory failure, hypotension, presence of fever, and renal impairment, for prediction of mortality was supported by several clinical studies [20, 26, 27, 39]. Although the statistical significance of age as a predictor for mortality was not identified in our data, it was included in our final model as, according to previous evidence [13, 25, 38], elderly was consistently reported as a marker of severity of acute illness and organ dysfunction during admission and a predictor of in-hospital mortality. For laboratory investigation, hypoalbuminemia was reported as a general marker for malnutrition and inflammation and was found to be associated with mortality during hospitalization in several patient populations [11, 39, 42, 43]. In AECOPD patients, the presence of hyponatremia, which can be caused by chronic salt-water retention due to common comorbidities of COPD (heart failure, Cor Pulmonale, adrenal insufficiency, or renal failure) or adverse effect of concomitant drug treatment), had significant impact on their morbidity and mortality [44].

In the reduced model, some pre-specified predictors were not included. Although previous studies reasonably supported radiographic consolidation (or the presence of pneumonia) [45], hyperglycemia [33], WBC count [39], and eosinophil count [13] as predictors for morbidity and mortality in AECOPD patients, their statistical significance could not be identified in our study, which might be explained by the relatively less discriminative contribution to the model in comparison to other relatively more significant predictors such as hypotension and requiring intubation. Without these predictors, the MAGENTA model still carried a good ability to differentiate AECOPD patients who would survive during their admission from patients who would not survive at an AuROC of 0.82. This good discriminative performance might be partially explained by appropriate fractional polynomial modeling of predictors and the incorporation of statistically significant predictors of mortality, which were pre-selected and supported by clinical studies as being associated with severity of acute illness and significant organ dysfunction. The drop in the discriminative ability of the MAGENTA model in the MICU subgroup might be due to the similarity in the severity of patients admitted to the ICUs. An entirely different set of predictors should be further explored if the prediction was performed only in a subgroup of patients admitted to the ICUs. However, as our study aimed to develop a prediction model for initial risk stratification of the AECOPD patients upon their admissions to general medical wards based on the predicted in-hospital mortality, the performance of the model in the MICU subgroup was irrelevant. In the subgroup of AECOPD patients admitted to general medical wards and were not subsequently transferred to the ICUs, the discriminative ability of the model was considered acceptable to excellent, suggesting that the MAGENTA model was appropriate for use in this intended to be prognosticated population.

Previously, several clinical prediction tools had been developed for the same purpose as the MAGENTA model; however, their predictive performance was only fair to good (AUROC 0.68 to 0.79) [25, 31, 34, 39, 46, 47]. Some of these prognostic scoring systems were primarily developed for use in other conditions, such as patients with community-acquired pneumonia (CURB-65 and CRB-65) [18, 47], derived from a cohort with a relatively smaller sample size [47], had a mixed population of COPD and asthma patients (COPD-asthma outcome study) [46], were limited to non-life-threatening exacerbation [25], and were conducted in the ICU setting [34]. CURB-65 was a well-validated and widely used scoring system for the prediction of patient mortality [18]. However, the predictive ability was only fair for patients with pneumonia (AuROC 0.73) [48] and even lower among AECOPD with pneumonia (AUROC 0.66) [49]. A simple score for predicting mortality in AECOPD patients, entitled DECAF, was developed in 2012 from five predictors, dyspnea based on the eMRCD scales, eosinopenia, consolidation, acidemia, and atrial fibrillation.

Although the DECAF score exhibited good to excellent discriminative ability at AUROC 0.86 [13] and was reported to be more accurate than the conventional CURB-65 [50, 51], the reliability and applicability of some predictors were still questionable, such as eMRCD scales and blood eosinophil level. In a developing country such as Thailand, eosinophilia can be caused by parasitic infestation or underlying atopic diseases [22], which threatened its reliability as a predictor within the DECAF score. Recently, one study in Thailand examined the association between eosinophilia and parasitic infestation in patients with COPD [52]. Although no intestinal parasite was identified in the study, and the authors suggested that the stool parasite exam might be omitted from routine COPD care, the sample size was small, and the study was conducted in the metropolitan region. Regarding current evidence, the eosinophil to guide treatment with management during AECOPD is still doubtful [53]. According to our data, the availability of arterial blood gas obtained within the first 24 hours of patient admissions was limited. In Thailand, an arterial puncture for blood gas analysis was not generally performed in non-intubated AECOPD patients. Another reason that hindered timely investigation of blood gas was a disproportion between the number of physicians available and the number of patients admitted.

The MAGENTA model carries several strengths over other clinical decision tools. Unlike others, the MAGENTA model incorporates seven objectives (i.e., simple laboratory parameters) and clinically available predictors (i.e., initial vital signs) to predict the individual probability of in-hospital mortality for each AECOPD patient. Predictors which were not routinely evaluated or collected were not included in the model, such as eMRCD scales, COPD severity based on spirometry results, and arterial blood gas results. Predictors with questionable inter-rater reliability, such as confusion status and consolidation on chest radiographs, were also omitted. Moreover, by utilizing fractional polynomial procedures, six continuous predictors were appropriately modelled according to the shape of predictor-outcome associations. In contrast, all previously reported clinical scores either dichotomized or categorized continuous predictors before modelling [25, 31, 34, 39, 46, 47]. Our approach preserved statistical power and improved model predictive accuracy.

For clinical applicability, the model predicted probabilities were subcategorized into three risk groups (low-risk, intermediate-risk, and high-risk) (Table 4). Recommendations for clinical management of each risk category were modified from the previous study [1]. For low-risk AECOPD patients, admission to the general ward is sufficient, and early patient discharge might be considered on a case-by-case basis. For intermediate-risk AECOPD patients, admission to the general ward is suggested. However, close monitoring of clinical progression is required, and the patients should be transferred to ICU if their symptoms deteriorated. For high-risk AECOPD patients, an urgent admission to ICU with early aggressive treatment is suggested. Nonetheless, the palliative care process might be initiated for patients with relatively high predicted probability, and unnecessary invasive procedures might be appropriately withheld.

The MAGENTA model still carries some limitations to be addressed. First, the model was derived from a retrospective observational cohort with incomplete data on clinically relevant factors (i.e., arterial blood gas, serum albumin, and point-of-care glucose testing). However, initial arterial blood gas was not usually investigated upon admission in all AECOPD patients, especially those who were not intubated. Thus, we did not include any component of arterial blood gas as one of our predictors. For serum albumin and point-of-care glucose, multiple imputation with chained equation was used during model derivation to account for the issue. Second, COPD diagnosis can only be validated in patients with available spirometry results, which accounts for only 30% of the cohort. This might threaten the validity of our model if all patients’ diagnoses could not be confirmed. However, this represents the real-world clinical practice where spirometry results were only available in less than 50% of COPD patients [20, 54]. Derivation of the prediction model from the cohort, which includes only patients with spirometry-confirmed AECOPD patients, might not be pragmatic. Although, the diagnosis of AECOPD in our study was based on clinician diagnosis by discharge summary. The finding of participant characteristics in our cohort described herein represents AECOPD because most patients were older men with a history of smoking similar to the previous report in north-eastern Thailand in 2014 [38]. Third, the MAGENTA model carries a relatively higher number of predictors than other decision tools; hence instead of presenting the model in a traditional perspective, the model was presented as a user-friendly, web-based calculator to enhance clinical applicability at the bedside. Fourth, the data used for developing the MAGENTA model was collected when NIV was limited and not routinely performed in patients with AECOPD. During the COVID-19 pandemic, many tertiary care hospitals, including ours, have a significantly increased NIV capacity. Therefore, acute management AECOPD patients with impending respiratory failure in our center might change after the pandemic, and the use of requiring intubation as a predictor within the MAGENTA model might not be entirely appropriate, and the MAGENTA model may require further modification to suit future practice. However, as NIV was still available in the tertiary care center and most patients with acute respiratory failure were still intubated and referred from the surrounding community hospitals, we believe that using requiring intubation as a predictor in this context is still practical. Finally, the development cohort was based on a single tertiary care hospital in Thailand, limiting the generalizability of the model to other settings. A prospective external validation study is required to confirm the reproducibility and transportability of the MAGENTA model prior to clinical implementation.

## Conclusion

The current prediction score, using the available routine parameters, had a good discriminative ability to differentiate between survived and non-survived admissions of AECOPD. The clinical implementation of the newly derived prediction score, MAGENTA, in resource-limited clinical settings could help clinicians make appropriate decisions in terms of the site of care, optimal monitoring, escalate/de-escalate treatment, and prognostic counselling.

## Supporting information

S1 Table Characteristics of patients in MICU and general wards. Comparison of clinical characteristics between AECOPD patients who were admitted to the MICU and patients who were admitted to the general medical wards (n = 923).
(DOCX)

S2 Table Characteristics of patients who were intubated and not intubated. Comparison of clinical characteristics between intubated AECOPD patients who were admitted to the MICU and patients who were admitted to the general medical wards (n = 657).
(DOCX)

S3 Table Sensitivity analysis result. Multivariable logistic regression model with exclusion of repeated admission records (n = 600).
(DOCX)
