# Supplementary material for: The MAGENTA model for individual prediction of in-hospital mortality in chronic obstructive pulmonary disease with acute exacerbation in resource-limited countries: A development study
Source: PLoS One. 2021 Aug 27;16(8):e0256866. doi: 10.1371/journal.pone.0256866 (PMC8396787; doi:10.1371/journal.pone.0256866)
Supplement: S1 Table — Comparison of clinical characteristics between AECOPD patients who were admitted to the MICU and patients who were admitted to the general medical wards (n = 923). (DOCX) [file pone.0256866.s001.docx]

**S1 Table. Characteristics of patients in MICU and general wards.** Comparison of clinical characteristics between AECOPD patients who were admitted to the MICU and patients who were admitted to the general medical wards (n=923)

| **Characteristics** | **Missing values**, n (%) | **MICU** (n=95) | | **Missing values**, n (%) | **General wards** (n=828) | | **p-value** |
| --- | --- | --- | --- | --- | --- | --- | --- |
|  |  | n | (%) |  | n | (%) |  |
| **Male**, (n, %) | 0 | 82 | (86.3) | 0 | 716 | (86.5) | 1.000 |
| **Age**, years, mean (±SD) | 0 | 76.6 | (10.4) | 0 | 74.1 | (11.2) | 0.043 |
| **Body mass index,** kg/m^2^, mean (±SD**)** | 45 (47.4) | 19.6 | (3.8) | 326 (39.4) | 20.4 | (4.4) | 0.220 |
| **Smoking status** | 7 (7.4) |  |  | 34 (4.1) |  |  | 0.740 |
| Never smoker, (n, %) |  | 7 | (7.4) |  | 59 | (7.1) |  |
| Ex-smoker, (n, %) |  | 72 | (75.8) |  | 630 | (76.1) |  |
| Active smoker, (n, %) |  | 9 | (9.5) |  | 105 | (12.7) |  |
| No. of cigarettes smoked, pack-year, median (IQR) | 37 (39.0) | 25.0 | (15.0, 40.0) | 273 (33.0) | 23.0 | (15.0, 40.0) | 0.970 |
| **Underlying diseases**, (n, %) |  |  |  |  |  |  |  |
| Present (any) | 0 | 78 | (82.1) | 0 | 659 | (79.6) | 0.690 |
| Hypertension | 0 | 40 | (42.1) | 0 | 353 | (42.6) | 1.000 |
| Diabetes mellitus | 0 | 9 | (9.5) | 0 | 119 | (14.4) | 0.210 |
| Ischemic heart disease | 0 | 16 | (16.8) | 0 | 93 | (11.2) | 0.130 |
| Atrial fibrillation | 0 | 11 | (11.6) | 0 | 48 | (5.8) | 0.043 |
| Left ventricular dysfunction | 0 | 2 | (2.1) | 0 | 20 | (2.4) | 1.000 |
| Chronic kidney disease | 0 | 7 | (7.4) | 0 | 72 | (8.7) | 0.850 |
| Cerebrovascular disease | 0 | 10 | (10.5) | 0 | 71 | (8.6) | 0.560 |
| Cognitive impairment | 0 | 1 | (1.1) | 0 | 14 | (1.7) | 1.000 |
| **COPD status** |  |  |  |  |  |  |  |
| FEV1/FVC ratio, mean (±SD) | 80 (84.2) | 0.5 | (0.1) | 616 (74.4) | 0.5 | (0.1) | 0.280 |
| FEV1, % predicted, mean (±SD) | 80 (84.2) | 35.9 | (17.0) | 616 (74.4) | 43.2 | (19.3) | 0.150 |
| FVC, % predicted, mean (±SD) | 80 (84.2) | 58.8 | (18.5) | 616 (74.4) | 66.4 | (20.4) | 0.160 |
| Long-term oxygen therapy, (n, %) | 0 | 4 | (4.2) | 0 | 83 | (10.0) | 0.092 |
| Cor pulmonale, (n, %) | 0 | 6 | (6.3) | 0 | 31 | (3.7) | 0.260 |
| **Initial vital signs** |  |  |  |  |  |  |  |
| Body temperature, ºC, mean (±SD) | 0 | 37.0 | (0.8) | 1 (0.1) | 37.1 | (0.6) | 0.130 |
| Heart rate, per minute, mean (±SD) | 0 | 108.5 | (21.7) | 0 | 99.8 | (19.4) | <0.001 |
| Systolic BP, mmHg, mean (±SD) | 0 | 128.0 | (30.3) | 0 | 133.3 | (23.5) | 0.043 |
| Diastolic BP, mmHg, mean (±SD) | 0 | 79.1 | (17.9) | 0 | 79.8 | (13.8) | 0.630 |
| Mean arterial pressure, mmHg, mean (±SD) | 0 | 95.3 | (20.3) | 0 | 97.6 | (15.7) | 0.190 |
| Respiratory rate, per minute, mean (±SD) | 0 | 24.9 | (5.2) | 0 | 24.7 | (4.4) | 0.650 |
| **Respiratory failure on admission (Intubation),** (n, %) | 0 | 91 | (95.8) | 0 | 566 | (68.4) | <0.001 |
| **Radiographic consolidation**, (n, %) | 0 | 32 | (33.7) | 0 | 337 | (40.7) | 0.220 |
| **Laboratory investigations** |  |  |  |  |  |  |  |
| Arterial blood gas |  |  |  |  |  |  |  |
| pH, mean (±SD) | 64 (67.4) | 7.2 | (0.2) | 684 (82.6) | 7.4 | (0.1) | <0.001 |
| PaO_2_, mmHg, median (IQR) | 67 (70.5) | 111.5 | (77.1, 182.5) | 687 (83.0) | 154.0 | (94.4, 259.0) | 0.024 |
| PaCO_2_, mmHg, median (IQR) | 64 (67.4) | 39.6 | (30.6, 59.7) | 684 (82.6) | 37.8 | (28.3, 45.5) | 0.170 |
| Sodium, mmol/l, mean (±SD) | 0 | 138.4 | (5.3) | 4 (0.5) | 138.7 | (5.1) | 0.600 |
| Potassium, mmol/l, mean (±SD) | 0 | 4.2 | (0.7) | 1 (0.1) | 4.0 | (0.7) | 0.037 |
| Chloride, mmol/l, mean (±SD) | 0 | 97.3 | (6.3) | 2 (0.2) | 97.6 | (6.0) | 0.670 |
| Bicarbonate, mmol/l, mean (±SD) | 0 | 23.6 | (5.6) | 1 (0.1) | 24.2 | (5.1) | 0.290 |
| Blood urea nitrogen, mg/dl, median (IQR) | 0 | 17.0 | (13.0, 23.0) | 0 | 16.0 | (11.0, 22.0) | 0.018 |
| Serum creatinine, mg/dl, median (IQR) | 0 | 1.1 | (0.8, 1.4) | 0 | 0.9 | (0.8, 1.2) | 0.003 |
| Serum albumin, g/dl, mean (±SD) | 27 (28.4) | 3.6 | (0.5) | 296 (35.8) | 3.9 | (0.5) | <0.001 |
| Complete blood count |  |  |  |  |  |  |  |
| Haemoglobin, g/dl, mean (±SD) | 0 | 12.5 | (1.7) | 1 (0.1) | 12.9 | (1.9) | 0.029 |
| WBC, /mm^3^, mean (±SD) | 0 | 14688.4 | (6328.5) | 1 (0.1) | 13819.1 | (6185.4) | 0.200 |
| Neutrophil, |  |  |  |  |  |  |  |
| count, /mm^3^, median (IQR) | 0 | 11075.0 | (7961.1, 16350.0) | 1 (0.1) | 10718.4 | (7395.0, 15009.6) | 0.390 |
| percent, mean (±SD) | 0 | 82.7 | (13.9) | 1 (0.1) | 83.4 | (13.7) | 0.630 |
| Eosinophil, median (IQR) |  |  |  |  |  |  |  |
| count, /mm^3^ | 0 | 14.9 | (0, 211.1) | 1 (0.1) | 35.4 | (0, 226.9) | 0.250 |
| percent | 0 | 0.1 | (0, 2.0) | 1 (0.1) | 0.3 | (0, 2.4) | 0.170 |
| Platelet count, /mm^3^, mean (±SD) | 0 | 247947.4 | (95959.1) | 1 (0.1) | 257221.3 | (90060.8) | 0.350 |
| Initial glucose, mg/dl, mean (±SD) | 19 (20.0) | 171.4 | (165.9) | 199 (24.0) | 153.9 | (60.5) | 0.069 |
| **Mechanical ventilator duration**, days, median (IQR) | 0 | 3.0 | (2.0, 7.0) | 0 | 2.0 | (0.0, 4.0) | <0.001 |
| **Length of hospital stay**, days, median (IQR) | 0 | 5.0 | (3.0, 9.0) | 0 | 4.0 | (2.0, 7.0) | 0.015 |

**Abbreviations:** AECOPD, acute exacerbation of chronic obstructive pulmonary disease; BP, blood pressure; FEV1, forced expiratory volume-one second; FVC, forced vital capacity; IQR, interquartile range; MICU, medical intensive care unit; SD, standard deviation; WBC, white blood cell count
