# Supplementary material for: The MAGENTA model for individual prediction of in-hospital mortality in chronic obstructive pulmonary disease with acute exacerbation in resource-limited countries: A development study
Source: PLoS One. 2021 Aug 27;16(8):e0256866. doi: 10.1371/journal.pone.0256866 (PMC8396787; doi:10.1371/journal.pone.0256866)
Supplement: S2 Table — Comparison of clinical characteristics between intubated AECOPD patients who were admitted to the MICU and patients who were admitted to the general medical wards (n = 657). (DOCX) [file pone.0256866.s002.docx]

**S2 Table. Characteristics of patients who were intubated and not intubated.** Comparison of clinical characteristics between intubated AECOPD patients who were admitted to the MICU and patients who were admitted to the general medical wards (n=657)

| **Characteristics** | **Missing values**, n (%) | **MICU** (n=91) | | **Missing values**, n (%) | **General wards** (n=566) | | **p-value** |
| --- | --- | --- | --- | --- | --- | --- | --- |
|  |  | n | (%) |  | n | (%) |  |
| **Male**, (n, %) | 0 | 79 | (86.8) | 0 | 501 | (88.5) | 0.600 |
| **Age**, years, mean (±SD) | 0 | 76.1 | (10.2) | 0 | 74.9 | (10.5) | 0.300 |
| **Body mass index,** kg/m^2^, mean (±SD**)** | 43 (47.3) | (19.4 | (3.8) | 261 (46.1) | 19.5 | (4.1) | 0.930 |
| **Smoking status** | 7 (7.7) |  |  | 29 (5.1) |  |  | 0.790 |
| Never smoker, (n, %) |  | 5 | (5.5) |  | 35 | (6.2) |  |
| Ex-smoker, (n, %) |  | 70 | (76.9) |  | 429 | (75.8) |  |
| Active smoker, (n, %) |  | 9 | (9.9) |  | 73 | (12.9) |  |
| No. of cigarettes smoked, pack-year, median (IQR) | 35 (38.5) | 25.0 | (15.0, 40.0) | 196 (34.6) | 21.3 | (12.5, 30.0) | 0.620 |
| **Underlying diseases**, (n, %) |  |  |  |  |  |  |  |
| Present (any) | 0 | 75 | (82.4) | 0 | 459 | (81.1) | 0.890 |
| Hypertension | 0 | 38 | (41.8) | 0 | 243 | (42.9) | 0.910 |
| Diabetes mellitus | 0 | 9 | (9.9) | 0 | 75 | (13.3) | 0.500 |
| Ischemic heart disease | 0 | 15 | (16.5) | 0 | 67 | (11.8) | 0.230 |
| Atrial fibrillation | 0 | 9 | (9.9) | 0 | 33 | (5.8) | 0.160 |
| Left ventricular dysfunction | 0 | 1 | (1.1) | 0 | 12 | (2.1) | 1.000 |
| Chronic kidney disease | 0 | 7 | (7.7) | 0 | 48 | (8.5) | 1.000 |
| Cerebrovascular disease | 0 | 10 | (11.0) | 0 | 57 | (10.1) | 0.850 |
| Cognitive impairment | 0 | 0 | (0) | 0 | 6 | (1.1) | 1.000 |
| **COPD status** |  |  |  |  |  |  |  |
| FEV1/FVC ratio, mean (±SD) | 76 (83.5) | 0.47 | (0.1) | 442 (78.1) | 0.48 | (0.1) | 0.486 |
| FEV1, % predicted, mean (±SD) | 76 (83.5) | 35.9 | (17.0) | 442 (78.1) | 41.9 | (18.2) | 0.230 |
| FVC, % predicted, mean (±SD) | 76 (83.5) | 58.8 | (18.5) | 442 (78.1) | 65.4 | (19.9) | 0.220 |
| Long-term oxygen therapy, (n, %) | 0 | 3 | (3.3) | 0 | 46 | (8.1) | 0.130 |
| Cor pulmonale, (n, %) | 0 | 6 | (6.6) | 0 | 15 | (2.7) | 0.057 |
| **Initial vital signs** |  |  |  |  |  |  |  |
| Body temperature, ºC, mean (±SD) | 0 | 37.0 | (0.8) | 0 | 37.2 | (0.6) | 0.050 |
| Heart rate, per minute, mean (±SD) | 0 | 108.9 | (21.6) | 0 | 100.2 | (19.4) | <0.001 |
| Systolic BP, mmHg, mean (±SD) | 0 | 127.1 | (30.4) | 0 | 132.0 | (23.8) | 0.078 |
| Diastolic BP, mmHg, mean (±SD) | 0 | 79.1 | (18.1) | 0 | 80.2 | (13.8) | 0.520 |
| Mean arterial pressure, mmHg, mean (±SD) | 0 | 95.1 | (20.6) | 0 | 97.5 | (15.9) | 0.200 |
| Respiratory rate, per minute, mean (±SD) | 0 | 24.7 | (4.9) | 0 | 24.0 | (3.6) | 0.078 |
| **Radiographic consolidation**, (n, %) | 0 | 31 | (34.1) | 0 | 263 | (46.5) | 0.031 |
| **Laboratory investigations** |  |  |  |  |  |  |  |
| Arterial blood gas |  |  |  |  |  |  |  |
| pH, mean (±SD) | 60 (65.9) | 7.2 | (0.2) | 440 (77.7) | 7.4 | (0.1) | <0.001 |
| PaO_2_, mmHg, median (IQR) | 63 (69.2) | 111.5 | (77.1, 182.5) | 442 (78.1) | 168.5 | (99.8, 266.5) | 0.008 |
| PaCO_2_, mmHg, median (IQR) | 60 (65.9) | 39.6 | (30.6, 59.7) | 440 (77.7) | 37.8 | (28.5, 45.1) | 0.170 |
| Sodium, mmol/l, mean (±SD) | 0 | 138.4 | (5.3) | 0 | 139.0 | (5.2) | 0.330 |
| Potassium, mmol/l, mean (±SD) | 0 | 4.2 | (0.7) | 0 | 4.1 | (0.7) | 0.280 |
| Chloride, mmol/l, mean (±SD) | 0 | 97.3 | (6.4) | 0 | 97.6 | (6.2) | 0.730 |
| Bicarbonate, mmol/l, mean (±SD) | 0 | 23.4 | (5.6) | 0 | 24.4 | (5.2) | 0.078 |
| Blood urea nitrogen, mg/dl, median (IQR) | 0 | 18.0 | (14.0, 24.0) | 0 | 17.0 | (12.0, 23.0) | 0.130 |
| Serum creatinine, mg/dl, median (IQR) | 0 | 1.1 | (0.8, 1.5) | 0 | 1.0 | (0.8, 1.2) | 0.011 |
| Serum albumin, g/dl, mean (±SD) | 25 (27.5) | 3.6 | (0.5) | 189 (33.4) | 3.8 | (0.5) | 0.003 |
| Complete blood count |  |  |  |  |  |  |  |
| Haemoglobin, g/dl, mean (±SD) | 0 | 12.5 | (1.7) | 1 (0.2) | 12.7 | (1.8) | 0.150 |
| WBC, /mm^3^, mean (±SD) | 0 | 14714.3 | (6411.8) | 1 (0.2) | 14654.2 | (6241.7) | 0.930 |
| Neutrophil, |  |  |  |  |  |  |  |
| count, /mm^3^, median (IQR) | 0 | 11075.0 | (7961.1, 16350.0) | 1 (0.2) | 12070.4 | (8500.8, 15793.2) | 0.400 |
| percent, mean (±SD) | 0 | 82.6 | (14.0) | 1 (0.2) | 85.5 | (12.4) | 0.038 |
| Eosinophil, median (IQR) |  |  |  |  |  |  |  |
| count, /mm^3^ | 0 | 14.6 | (0, 211.1) | 1 (0.2) | 30.8 | (0, 211.8) | 0.430 |
| percent | 0 | 0.1 | (0, 2.0) | 1 (0.2) | 0.2 | (0, 2.0) | 0.440 |
| Platelet count, /mm^3^, mean (±SD) | 0 | 247670.3 | (97940.5) | 1 (0.2) | 256923.9 | (91031.4) | 0.370 |
| Initial glucose, mg/dl, mean (±SD) | 17 (18.7) | 172.1 | (167.9) | 86 (15.2) | 152.0 | (56.9) | 0.047 |
| **Mechanical ventilator duration**, days, median (IQR) | 0 | 4.0 | (2.0, 8.0) | 0 | 3.0 | (2.0, 6.0) | 0.600 |
| **Length of hospital stay**, days, median (IQR) | 0 | 5.0 | (3.0, 9.0) | 0 | 4.0 | (3.0, 8.0) | 0.420 |

**Abbreviations:** AECOPD, acute exacerbation of chronic obstructive pulmonary disease; BP, blood pressure; FEV1, forced expiratory volume-one second; FVC, forced vital capacity; IQR, interquartile range; MICU, medical intensive care unit; SD,
