# Supplementary material for: The MAGENTA model for individual prediction of in-hospital mortality in chronic obstructive pulmonary disease with acute exacerbation in resource-limited countries: A development study
Source: PLoS One. 2021 Aug 27;16(8):e0256866. doi: 10.1371/journal.pone.0256866 (PMC8396787; doi:10.1371/journal.pone.0256866)
Supplement: S3 Table — Multivariable logistic regression model with exclusion of repeated admission records (n = 600). (DOCX) [file pone.0256866.s003.docx]

**S3 Table. Sensitivity analysis result.** Multivariable logistic regression model with exclusion of repeated admission records (n=600).

|  | Full multivariable model | | | | Reduced multivariable model | | | |
| --- | --- | --- | --- | --- | --- | --- | --- | --- |
|  | **ß** | **95% CI** | | **P-value** | **ß** | **95% CI** | | **P-value** |
| **Demographic data** |  |  |  |  |  |  |  |  |
| FP1 Age, years | 0.018 | -0.012 | 0.049 | 0.230 | 0.015 | -0.0140 | 0.045 | 0.303 |
| **Initial assessments** |  |  |  |  |  |  |  |  |
| FP1 BT, ºC | 0.550 | 0.172 | 0.929 | 0.004 | 0.569 | 0.193 | 0.946 | 0.003 |
| FP1 MAP, mmHg | 0.880 | 0.360 | 1.400 | 0.001 | 0.899 | 0.366 | 1.432 | 0.001 |
| Require intubation | 2.375 | 1.039 | 3.711 | <0.001 | 2.219 | 0.939 | 3.499 | 0.001 |
| **Initial investigations** |  |  |  |  |  |  |  |  |
| Radiographic consolidation | 0.251 | -0.394 | 0.895 | 0.446 |  | Not included | | |
| **Complete blood count** |  |  |  |  |  |  |  |  |
| FP1 WBC count, /mm^3^ | 0.000 | -0.000 | 0.000 | 0.334 |  | Not included | | |
| FP1 Eosinophil count, /mm^3^ | 0.000 | -0.000 | 0.001 | 0.061 |  | Not included | | |
| **Blood chemistry** |  |  |  |  |  |  |  |  |
| FP1 Na, mmol/l | -0.080 | -0.130 | -0.030 | 0.002 | -0.073 | -0.121 | -0.024 | 0.003 |
| FP1 BUN, mg/dl | -21.116 | -38.472 | -3.760 | 0.017 | -21.352 | -37.894 | -4.810 | 0.011 |
| FP2 BUN, mg/dl | -51.537 | -86.352 | -16.721 | 0.004 | -54.331 | -85.098 | -23.565 | 0.001 |
| FP1 SCr, mg/dl | 0.278 | -0.318 | 0.8747 | 0.361 |  | Not included | | |
| FP1 Serum albumin, g/dl | -1.034 | -1.712 | -0.356 | 0.003 | -1.037 | -1.678 | -0.397 | 0.002 |
| Constant | -4.905 | -6.302 | -3.507 |  | -4.609 | -5.905 | -3.313 |  |
| AuROC | 0.839 | 0.783 | 0.894 |  | 0.834 | 0.778 | 0.890 |  |
|  |  |  |  |  |  |  |  |  |

**Abbreviations:** AuROC, area under the receiver operating characteristic curve; ß, beta-coefficient (log odds ratio); BT, body temperature; BUN, blood urea nitrogen; CI, confidence interval; FP1, first-degree fractional polynomial transformation; FP2, second-degree fractional polynomial transformation; MAP, mean arterial pressure; NA, not applicable; Na, serum sodium; SCr, serum creatinine; WBC, white blood cell.
